# Performance of the systemic lupus erythematosus risk probability index (SLERPI) in the Egyptian college of rheumatology (ECR) study cohort

**DOI:** 10.1007/s10067-024-07210-0

**Published:** 2024-11-04

**Authors:** Nevin Hammam, Ahmed Elsaman, Esam Abualfadl, Soha Senara, Nada M. Gamal, Mona H. Abd-Elsamea, Abdelhfeez Moshrif, Osman Hammam, Tamer A. Gheita, Samar Tharwat, on behalf of the Egyptian College of Rheumatology (ECR) Study Group

**Affiliations:** 1 Rheumatology Department, Faculty of Medicine, Assiut University, Assiut, Egypt; 2 Rheumatology Department, Faculty of Medicine, Sohag University, Sohag, Egypt; 3 Qena/Luxor Hospitals, Qena, Egypt; 4 Rheumatology Department, Faculty of Medicine, Fayoum University, Fayoum, Egypt; 5 Rheumatology Department, Faculty of Medicine, Al-Azhar University, Assuit, Egypt; 6 Department of Rheumatology and Rehabilitation, Faculty of Medicine, New Valley University, New Valley, Egypt; 7 Rheumatology Department, Faculty of Medicine, Cairo University, Cairo, Egypt; 8 Rheumatology Unit, Internal Medicine, Mansoura University Hospital, El Gomhouria St, Mansoura, Dakahlia Governorate Egypt; 9 Department of Internal Medicine, Faculty of Medicine, Horus University, New Damietta, Egypt

**Keywords:** Classification criteria, SLE, SLE risk probability index

## Abstract

**Objectives:**

This study aimed to evaluate the performance of systemic lupus erythematosus Risk Probability Index (SLERPI) in Egyptian patients with SLE using a national rheumatology database.

**Methods:**

The Egyptian College of Rheumatology (ECR) database comprised of 1,162 patients with SLE and 4,327 with miscellaneous rheumatological diseases who were recruited from the Rheumatology Departments across the country. The diagnosis of SLE was established by expert rheumatologists. Variables of the SLERPI were extracted and recorded as present or absent for each patient. The absolute value for the SLERPI score was calculated for each patient, and the diagnosis of SLE was accounted for if the score was greater than 7 points.

**Results:**

Of 1,162 SLE patients evaluated, 1,031 (88.7%) patients were diagnosed with SLE according to the SLERPI, with an average score of 13.1 (3.8). Differences in the 14 SLERPI variables were significant between the SLE-SLERPI groups, except for the presence of leukopenia and positive ANA. As a score reduction item, the SLE-SLERPI > 7 group had lower interstitial lung diseases. Patients diagnosed with SLE according to SLERPI had significantly higher disease activity (*p* < 0.001), and this group more commonly received corticosteroids and mycophenolate mofetil. Compared to other miscellaneous rheumatological groups, all 14 SLERPI items are indeed more common in the SLE group. In terms of the overall performance of SLERPI in the diagnosis of SLE, the accuracy of SLERPI was 91.9% (95% CI 90.9%—92.9%), with a specificity of 96.95% and sensitivity of 86.9%. SLERPI showed that accuracy went up to 93.3% (95%CI 92.4%-94.2%), with a specificity of 94.9% and a sensitivity of 91.6% when patients with connective tissue diseases were taken out of the study.

**Conclusion:**

Using a large cohort of SLE, the SLERPI revealed excellent diagnostic efficacy and specificity. The use of SLERPI in clinical practice may contribute to improved patient diagnosis and prognosis.

**Table:** 

**Key Points** • *SLERPI's performance has high diagnostic efficiency in Egyptian SLE patients.* • *SLERPI score can efficiently distinguish patients with SLE from other CTDs.* • *Within the SLERPI score, interstitial lung disease is the lowest predictor of SLE.*

**Supplementary Information:**

The online version contains supplementary material available at 10.1007/s10067-024-07210-0.

## Introduction

Systemic lupus erythematosus (SLE) is a chronic autoimmune disease that affects the entire body and varies in its severity and progression that is characterized by recurring episodes of flare [1]. The clinical manifestations of SLE vary from mild cutaneous symptoms to severe organ failure [2]. The early manifestations of systemic lupus erythematosus are often vague and mimic other medical conditions, which heightens the likelihood of delayed diagnosis [3]. Early diagnosis and subsequent disease management are made more challenging by the heterogeneity of clinical manifestations, which can also delay the onset of effective treatment [4]. The diagnosis of SLE is therefore frequently delayed, resulting in irreversible organ damage by the time a formal diagnosis is confirmed [5]. From the 1982 and 1997 revised ACR criteria to the SLICC and, most recently, the EULAR/ACR 2019 criteria, SLE classification criteria have evolved as scientific tools for better studying SLE [6]. Despite the great progress in SLE classification criteria, they continue to have limitations and fail to classify most patients with early SLE. This necessitated the development of new criteria for the classification of SLE [7].

Machine learning (ML) employs algorithms, methodologies, and procedures to identify latent correlations in data and develop descriptive or predictive tools that exploit those associations [8]. Various research has utilized ML to detect individuals with rheumatic and autoimmune diseases, while others have utilized ML approaches to extract important clinical characteristics and classify patients [9–11].

Adamichou et al. [12] recently created a new ML-based model called the SLE Risk Probability Index (SLERPI) to assist in the early detection and treatment of SLE. This model utilizes 14 clinical and serological parameters, which are weighted differently depending on their relevance to the disease. These variables include cutaneous features, arthritis, serositis, neurological disorders, interstitial lung disease, hematological and immunological variables. Thrombocytopenia/haemolytic anaemia, malar/ maculopapular rash, proteinuria, low C3 and C4, antinuclear antibodies (ANA) and immunologic disorder being the strongest SLE predictors. When used as a dichotomous algorithm (SLE-or-not), the SLERPI score at least of 7 points exhibits high accuracy for SLE, including early and severe/organ-threatening disease forms. The sensitivity, specificity, and accuracy of this model in the European population were calculated to be 94.2%, 94.4%, and 94.2%, respectively [13]. Another study conducted in China indicated that this model exhibits a high level of sensitivity (98.3%) but a relatively low level of specificity (89.4%) [14]. The SLERPI scale has shown to exhibit higher accuracy than other clinical scales including the American college of rheumatology /European league against rheumatism (ACR/EULAR) or Systemic Lupus International Collaborating Clinics (SLICC) classification criteria [15, 16]. SLERPI could help in the early diagnosis and treatment of SLE, including its early and severe manifestations, to enhance patient outcomes [12].

Further validation of SLERPI in other races is necessary due to variations in diagnostic performance using existing classification criteria for diagnosing patients with SLE in different racial groups. Therefore, this study aimed to evaluate the performance of SLERPI in patients with SLE using a population-based, multicenter, cross-sectional Egyptian College of Rheumatology (ECR) cohort.

## Patients and methods

### Study design and participants

This is a retrospective analysis of the ECR cohort. ECR data were collected during routine clinical care from 2021–2022. The Egyptian cohort included 1,162 patients with SLE and 4,327 patients with miscellaneous rheumatological diseases, serving as a control group. These patients were recruited from multiple specialized rheumatology centers and departments all over Egypt by the members of the Egyptian Colleague of Rheumatology (ECR) study group [17] between 2018 and 2021. The diagnosis of SLE according to the SLICC classification criteria [18] was established by expert rheumatologists. Patients with other rheumatic diseases were diagnosed using standard criteria and confirmed by expert rheumatologists.

### Ethical consideration

The study was conducted in accordance with the principles indicated in the Helsinki Declaration [19], and the Institutional Research Board of the Faculty of Medicine at Mansoura University approved the study protocol (Approval No. R.24.07.2699) prior to its execution. Informed written consent was obtained from all participants. Enrollment in the study was completely optional and always maintained strict confidentiality.

### Data collection and variables

Sociodemographic data such as age and sex, as well as disease duration, age at the diagnosis of rheumatic disease, and the presence of comorbidities such as diabetes mellitus or hypertension, were reported. Disease-specific clinical and laboratory data were extracted from the database. Therapeutic data, such as systemic steroids, hydroxychloroquine, cyclophosphamide, azathioprine, mycophenolate mofetil, and methotrexate (MTX), were recorded.

### SLE subset collection

Each SLE patient underwent a comprehensive clinical assessment, specifically any clinical manifestations of SLE. The laboratory data that was considered important for analysis included the complete blood count, urine analysis, complement (C3 and C4) levels, and the autoantibody profile, which consisted of anti-nuclear antibody (ANA) and anti-double-stranded deoxyribonucleic acid (anti-dsDNA). Subsequently, the disease's activity level was evaluated using the SLE Disease Activity Index (SLEDAI) [20]. The Systemic Lupus International Collaborating Clinics/American College of Rheumatology (SLICC/ACR) Damage Index score (SDI) was computed for every SLE patient [21].

### Definition of the control population

Participants in the control group include patients with rheumatoid arthritis (*n* = 2,556), Behcet’s disease (*n* = 1,526), spondyloarthritis (*n* = 188), idiopathic inflammatory myopathies (*n* = 13), primary systemic vasculitis (*n* = 11), undifferentiated connective tissue disease (CTD) (*n* = 3), primary Sjogren’s syndrome (*n* = 7), systemic sclerosis (*n* = 2), and mixed CTD (*n* = 3). The diagnosis of miscellaneous control rheumatological diseases was made by consultant rheumatologists. The relevant information for each participant in the control group, such as demographics, clinical features, medication use, and applicable laboratory variables, was collected.

### SLERPI variables

The SLERPI model comprises 14 clinical and serological characteristics with varying weights [12]. The model incorporated characteristics from all three sets of classification criteria, as well as interstitial lung disease as a distinct non-criteria feature. Autoimmune thrombocytopenia or hemolytic anemia, malar or maculopapular rash, low levels of C3 and C4 proteins, presence of protein in urine (as defined by the EULAR/ACR 2019 criteria), presence of ANA, and the ACR 1997 immunological disease (updated to add anti-β2-glycoprotein antibodies). Based on the SLERPI score, participants were divided into two groups: the first group had a SLERPI score greater than 7, while the second group had a SLERPI less than or equal to 7.

## Statistical analysis

Statistical analyses were performed using Stata statistical software version 15 (Stata-Corp). Descriptive statistics are presented either as median and interquartile range (IQR) or mean and standard deviation (SD), as indicated. Categorical variables are presented as absolute values and percentages. The Mann–Whitney U-test or Student’s t-test was used for comparison of continuous variables, according to normality of distribution. For the evaluation of categorical variables, the Pearson’s chi-square test and Fisher’s final test were used, as indicated. For all comparisons, P-values < 0.05 were considered statistically significant. The performance of the SLERPI was evaluated by the receiver operating characteristic curve (AUC) accuracy, sensitivity, and specificity.

## Results

The sample consisted of 1,162 patients in the SLE group and 4,327 non-SLE patients in the control group. Compared with the control group, the SLE group consisted of younger patients and a higher proportion of women (Table 1). The median age of disease diagnosis was significantly lower in the SLE group compared to the control group (25 years vs. 35 years, *p* < 0.001).

**Table 1:** Evaluation of patients in the SLE group and control group according to SLERPI

Variable mean ± SD, *n* (%), median (IQR)	SLE group (*n* = 1,162)	Control group (*n* = 4,327)	P value
Age, years	33.3 ± 10.1	40.5 ± 11.7	< 0.001
Female	1,039 (89.4)	2,822 (65.2)	< 0.001
Diagnosis duration, years	4 (2,7)	5 (2,8)	0.179
Age of diagnosis	25 (20, 32)	34 (26, 42)	< 0.001
SLERPI variables			
Malar rash or maculopapular rash	676 (58.2)	405 (9.4)	< 0.001
SCLE or DLE	251 (21.6)	2 (0.05)	< 0.001
Alopecia	584 (50.3)	143 (3.3)	< 0.001
Mucosal ulcers	715 (61.5)	1,612 (37.2)	< 0.001
Arthritis	757 (65.1)	1,492 (34.5)	< 0.001
Serositis	219 (18.8)	41 (0.95)	< 0.001
Leucopenia	238 (20.5)	150 (4.2)	< 0.001
Thrombocytopenia or AIHA	154 (13.2)	127 (2.9)	< 0.001
Neurological disorder	127 (10.9)	220 (5.1)	< 0.001
Proteinuria	427 (36.7)	122 (2.8)	< 0.001
ANA	1,023 (88.0)	347 (8.0)	< 0.001
Low C3 and C4	239 (20.6)	8 (0.20)	< 0.001
Immunological disorder	501 (43.1)	15 (0.37)	< 0.001
Interstitial lung disease	66 (5.68)	11 (0.39)	< 0.001
Medications			
Systemic steroids	648 (89.2)	2,687 (63.4)	< 0.001
Hydroxychloroquine	588 (79.1)	1,608 (37.9)	< 0.001
Cyclophosphamide	226 (30.4)	226 (5.7)	< 0.001
Azathioprine	333 (44.8)	533 (12.6)	< 0.001
Mycophenolate mofetil	66 (10.2)	20 (0.47)	< 0.001
Methotrexate	98 (15.7)	1,854 (43.7)	< 0.001

*AIHA* autoimmune hemolytic anemia, *ANA* anti-nuclear antibodies, *C3* complement 3, *C4* complement 4, *SLERPI* Systemic Lupus Erythematosus Risk Probability Index. *DLE* discoid lupus erythematosus, *SCLE* Subacute cutaneous lupus erythematosus

Of the 14 SLERPI variables, all items are indeed more common in the SLE group than the control group. In the SLE group, most individuals (58.2%) exhibited either malar rash or maculopapular rash, compared to 9.4% in the control group (Table 1). Upon comparing the therapeutic data of the SLE group and the control group, the SLE group had a significantly higher prescription rate of systemic steroids, hydroxychloroquine, cyclophosphamide, azathioprine, and mycophenolate mofetil. On the other hand, the prescription rate of MTX was significantly lower in the SLE group (15.7% vs. 43.7%, *p* < 0.001).

**Fig. 1:**
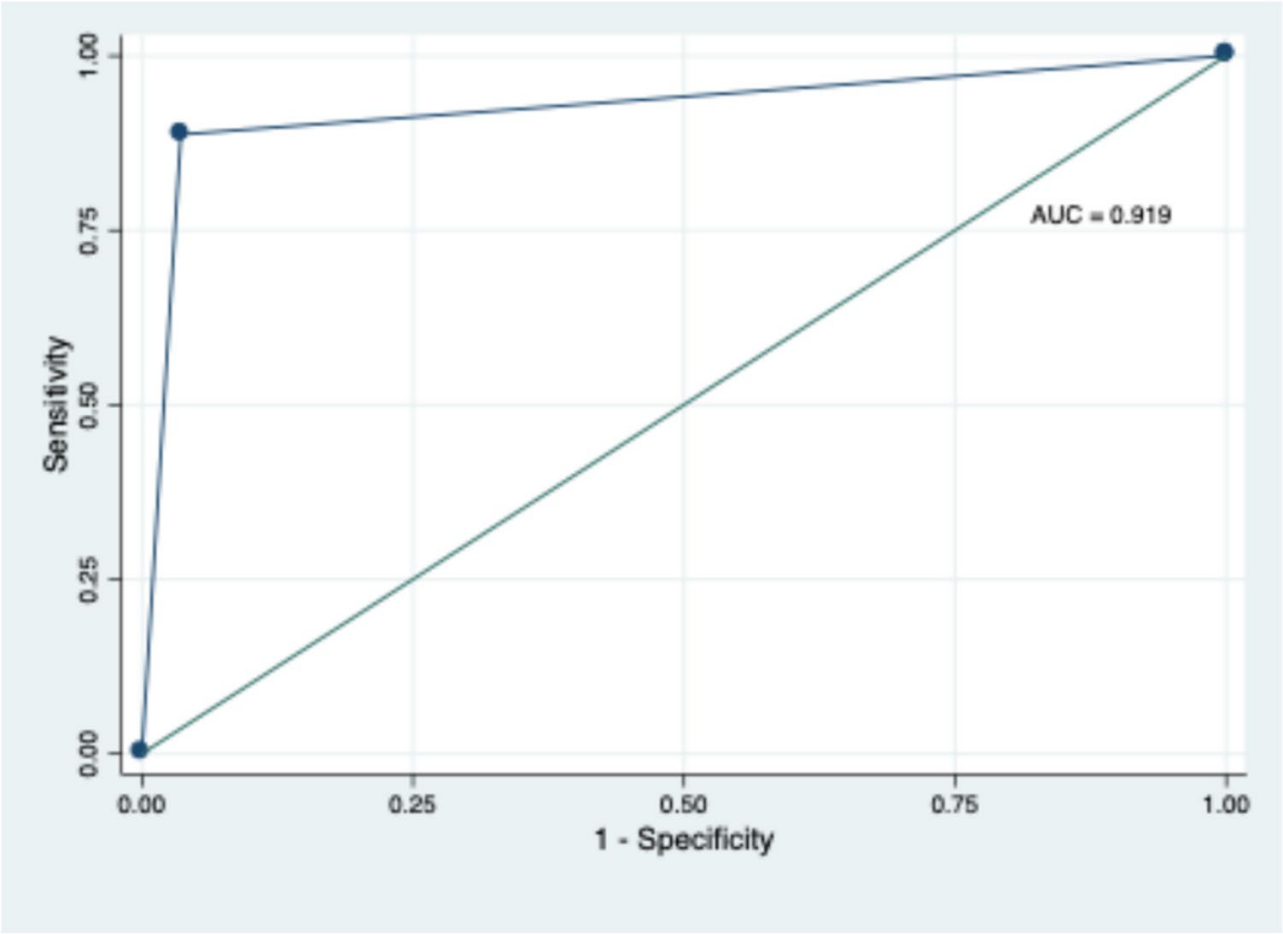
The receiver operating curve with a calculated area under the curve indicating an excellent capacity of the SLERPI to discriminate SLE versus miscellaneous rheumatology disease controls

In terms of the overall performance of SLERPI in the diagnosis of SLE, the accuracy of SLERPI was 91.9% (95% CI: 90.9–92.9), with a specificity of 96.9% and sensitivity of 86.9% (Fig. 1).

Among 1,162 SLE patients evaluated, 1,031 (88.7%) patients were diagnosed with SLE according to the SLERPI scale (> 7 points). Age, gender, duration, age at SLE diagnosis, and presence of comorbidities did not exhibit any statistically significant differences between individuals with SLERPI > 7 and those with SLERPI ≤ 7. The average score in patients classified with SLE using the SLERPI scale was 13.1 (3.8), as demonstrated in Table 2. The SLE group with SLERPI > 7 exhibited a statistically significantly higher SLEDAI score (*p* < 0.001) and a higher frequency of corticosteroids and mycophenolate mofetil users. Nevertheless, the frequency of MTX administration was lower in this group (10.95% vs. 25%, p = 0.001). Of the 14 SLERPI items, differences in the variables were significant between the SLE-SLERPI > 7 and SLE-SLERPI group ≤ 7 groups, except for the presence of leukopenia and positive ANA (Table 2). As a score reduction item, the SLE-SLERPI > 7 group had a lower prevalence of interstitial lung disease.

**Table 2:** Comparison of clinical characteristics of patients with SLE risk probability score > 7 and ≤ 7

Variable mean ± SD, *n* (%), median (IQR)	SLE risk probability > 7 (*n* = 1,031)	SLE risk probability ≤ 7 (*n* = 130)	P value
Age, years	33.1 ± 10.2	34.8 ± 9.4	0.069
Female	920 (89.2)	119 (90.8)	0.574
Diagnosis duration, years	4 (2,7)	5.5 (3,9)	0.576
Age of diagnosis	26.5 (8.4)	28.1 (9.0)	0.129
Diabetes	9 (25.7)	148 (35.7)	0.236
Hypertension	3 (12.5)	35 (9.7)	0.656
SLERPI variables			
Malar rash or maculopapular rash	664 (64.4)	12 (9.16)	< 0.001
SCLE or DLE	240 (23.3)	11 (8.4)	< 0.001
Alopecia	563 (54.6)	21 (16.0)	< 0.001
Mucosal ulcers	715 (61.5)	0 (0.0)	NA
Arthritis	696 (67.5)	61 (46.5)	< 0.001
Serositis	209 (20.3)	10 (7.6)	< 0.001
Leucopenia	219 (21.2)	19 (14.5)	0.072
Thrombocytopenia or AIHA	154 (14.9)	0 (0.0)	NA
Neurological disorder	120 (11.6)	7 (5.3)	0.030
Proteinuria	427 (41.4)	0 (0.0)	NA
ANA	912 (88.5)	111 (84.7)	0.216
Low C3 and C4	232 (22.5)	7 (5.3)	< 0.001
Immunological disorder	480 (46.5)	21 (16.0)	< 0.001
Interstitial lung disease	58 (5.6)	8 (6.1)	0.823
SLERPI score	13.1 ± 3.8	5.6 ± 1.2	< 0.001
SLEDAI score	11 (4—20)	4 (1—8)	< 0.001
SLICC -DI	2 (1- 3)	1 (0—1)	0.228
Medications			
Systemic steroids	520 (88.6)	56 (81.2)	0.075
Hydroxychloroquine	509 (86.7)	63 (91.3)	0.280
Cyclophosphamide	215 (36.6)	25 (36.2)	0.949
Azathioprine	297 (50.6)	35 (50.7)	0.984
Mycophenolate mofetil	64 (13.1)	4 (5.8)	0.083
Methotrexate	51 (10.9)	17 (25.0)	0.001

*AIHA* autoimmune hemolytic anemia, *ANA* anti-nuclear antibodies, *C3* complement 3, *C4* complement 4, *SLEDAI* Systemic Lupus Erythematosus Disease Activity Index, *SLERPI* Systemic Lupus Erythematosus Risk Probability Index. *SLICC-DI* Systemic Lupus Erythematosus International Collaborating Clinics damage index, *DLE* discoid lupus erythematosus, *SCLE *Subacute cutaneous lupus erythematosus

## Discussion

Despite significant advancements in diagnosis and treatment strategies that have improved the prognosis of SLE, there remain many unfulfilled requirements for SLE diagnosis [22]. Implementing effective tools for early SLE diagnosis presents a significant challenge. If a window of opportunity for SLE patients is identified and shown to improve outcomes, such as damage, death, recurring flares, and health-related quality of life measurements, then these would become even more valuable [23].

SLERPI, a novel and straightforward model, uses commonly observed clinical and serological characteristics to detect SLE. This model offers risk predictions that are strongly associated with clinical outcomes and can assist in classifying patients based on the probability of developing a clinically significant disease. The SLERPI has the potential to enhance patient outcomes by facilitating the diagnosis and treatment of SLE, including early and severe forms [13]. This new model has the capacity to generate individualized risk probabilities for SLE, which allows for the classification of a validation cohort into unlikely, possible, probable, and definite SLE. Using SLERPI in clinical practice could help patients with SLE receive more timely therapies and improve their prognosis [14]. Like other recent criteria, the SLERPI criteria put an emphasis on having an acute malar rash, hematological symptoms (such as autoimmune hemolytic anemia or thrombocytopenia), and proteinuria [12]. Further validation of SLERPI in various races is necessary due to variations in diagnostic performance using existing classification criteria for diagnosing individuals with SLE [24, 25]. The current study evaluated SLERPI's performance in a large cohort of Egyptians and found that it has high diagnostic efficiency.

The current cohort showed that almost all SLERPI variables were more common in the SLE group compared to the control group. In the SLE group, more than half individuals (58.2%) exhibited either malar rash or maculopapular rash. In this regard, about half of SLE patients have malar rash at the time of diagnosis, which might occur weeks or months before systemic involvement manifests [26]. We also observed SCLE or discoid lupus erythematosus (DLE) in 21.6% of the SLE group, compared to 0.05% in the control group. SCLE is typically a photosensitive skin condition characterized by a non-scarring, non-atrophy-inducing, red circular or scaly rash [27]. However, SCLE may also be found in patients with primary Sjögren's syndrome, rheumatoid arthritis, temporal arteritis, dermatomyositis, and ankylosing spondylitis [28].

In the current study, approximately 62% of SLE individuals experienced mucosal ulcers, compared to 37.2% in the control group. Typically, approximately 50% of patients with SLE develop mucosal ulcers [29]. The tongue, oral mucosa, lips, and palate are the primary areas affected by SLE. Because of this, oral ulcers are considered primary events, and they are included in almost all SLE activity [20, 30, 31].

We observed alopecia in 50.3% of SLE patients in our cohort, compared to only 3.3% in the control group. Alopecia affects 40–70% of individuals with SLE, resulting in widespread hair thinning and fragility caused by telogen/anagen effluvium and lupus hairs. This leads to generalized thinning or a receding frontal hairline with broken hairs. The 2012 SLICC classification for SLE includes alopecia as one of its criteria [32]. The SLICC criteria specifically include non-scarring alopecia because of its high specificity to SLE (95.7%) in the derivation sample, and it also meets the standards of clinical consensus among experts [33].

In the SLE group, arthritis was the most common SLERPI clinical manifestation, accounting for 65.1% of cases. Conti F and coauthors found that musculoskeletal manifestations were present in as many as 80% of SLE patients. SLE arthritis is mostly characterized by symmetric involvement, predominantly affecting the small joints [34]. The significance of joint involvement in SLE led to its inclusion in the ACR classification criteria, which were proposed in 1982 [35]. It is important to note that the various criteria define joint involvement in different ways. Joint involvement is defined by the 1982 ACR criteria and the revised version that came out in 1997 as "non-erosive arthritis affecting two or more peripheral joints, characterized by tenderness, swelling, or effusion" [35, 36]. Nevertheless, the 2012 SLICC criteria expand the concept of joint involvement to include arthralgia accompanied by stiffness [18].

When we compared the therapeutic data of the SLE group and the control group, we noticed that the prescription rate of MTX was significantly lower in the SLE group. MTX is typically effective in managing musculoskeletal, cutaneous, and serosal conditions in SLE patients [37]. MTX may not be required for patients with mild SLE who are effectively managed on hydroxychloroquine and may not be suitable for those with severe disease who require more intensive treatments. MTX is considered an option for the majority of patients with non-organ-threatening disease [37]. European guidelines for cutaneous lupus erythematosus advocated the use of MTX as the second line of systemic treatment after the failure of antimalarials [38].

In terms of the overall performance of SLERPI in the diagnosis of SLE; the accuracy of SLERPI was 91.9%, with a specificity of 96.9% and sensitivity of 86.9%. We further tested the performance of this SLERPI after excluding patients with connective tissue diseases (supplementary Fig. 2). The results showed that accuracy improved 93.3% (95%CI 92.4%-94.2%), with a specificity of 94.9% and sensitivity of 91.6%. Several studies have indicated that the SLERPI scale exhibits a sensitivity ranging from 88.8% to 97.6% for the diagnosis of SLE and demonstrated superior performance compared to other clinical scales such as ACR/EULAR or SLICC [15, 39]. The SLERPI scale demonstrates superiority over other measures in the identification of SLE in undifferentiated connective tissue disease, which may facilitate early management and potentially reduce complications [16]. SLERPI should be utilized in newly diagnosed and incident patients rather than prevalent cases as it is a predictive tool.

The SLERPI model demonstrated a strong combination of sensitivity (95.1%) and specificity (93.7%) when SLERPI was considered as binary—that is, SLE or non-SLE. Also, including early SLE patients, SLICC 2012 and SLERPI 2020 exhibited rather good sensitivity at 97.6%. With the ACR 1997 criteria, the specificity remained the highest. SLERPI might thus be helpful for early identification and therapy of SLE patients [40]. Subject to additional validation in various settings, the SLERPI has the potential to serve as a screening tool for individuals displaying non-specific serological characteristics (such as isolated ANA) along with significant clinical symptoms (such as malar rash or proteinuria > 500 mg/24 h). It may also be useful for individuals experiencing multiple clinical symptoms without any immunological abnormalities or when specific autoantibodies (such as anti-dsDNA, anti-Sm) coincide with a single clinical feature (such as thrombocytopenia or autoimmune hemolytic anemia) [41].

A noteworthy observation was made during our analysis of the SLERPI score in the study population. Of 1,162 SLE patients evaluated, 1,031 (88.7%) patients were diagnosed with SLE according to the SLERPI scale. In the "Attikon" SLE cohort, an observational study of 855 patients, 87.4% of patients had an SLERPI > 7 at the time of hospitalization. According to the authors, the SLERPI demonstrated a slight increase in diagnostic accuracy for SLE; however, the rates were lower than anticipated due to the absence of clinical manifestations, such as fever and thrombosis, in this scoring system. The likelihood of a correct diagnosis was increased by modifying SLERPI to a lower cut-off (threshold of 5) in hospitalized patients who presented with fever and thrombotic events [39].

In our cohort, the SLE group with SLERPI > 7 exhibited a statistically significantly higher SLEDAI score. The SLEDAI and its modified version, SLEDAI-2 K, are widely utilized disease activity indices for SLE in clinical settings [42]. In this regard, it is documented that patients with higher EULAR/ACR-2019 scores exhibit higher disease activity indices in SLE [43].

To the best of our knowledge, this is the first study that evaluates the performance of SLERPI among Egyptian SLE patients. An important strength of this study was the large sample size. Also, our results enrich the knowledge of early SLE prediction, thereby contributing to the understanding of the disease and its management. It is necessary to realize that this study has several limitations. The study was conducted on an Egyptian population, so the results may not be applicable to all individuals with SLE. In addition, although SLE is not uncommon in childhood, we primarily assessed adult patients, suggesting that further studies in this patient subset are required.

## Conclusion

Using a large cohort of SLE, the SLERPI revealed excellent diagnostic efficacy and specificity. The use of SLERPI in clinical practice may contribute to improved patient diagnosis and prognosis. However, additional research with different ethnicities is required to verify the performance of SLERPI in detecting SLE.

## Supplementary information

Below is the link to the electronic supplementary material.

Supplementary file1 (DOCX 82 KB)

## Data Availability

The datasets used and/or analysed during the current study are available from the corresponding author on reasonable request.

## Abbreviations

ANA: Anti-nuclear antibody
Anti-dsDNA: Anti-double-stranded deoxyribonucleic acid
DLE: Discoid lupus erythematosus
ECR: Egyptian College of Rheumatology
ML: Machine learning
SCLE: Subacute cutaneous lupus erythematosus
SDI: Damage Index score
SLE: Systemic lupus erythematosus
SLEDAI: SLE Disease Activity Index
SLERPI: Systemic lupus erythematosus risk probability index
SLICC/ACR: Systemic Lupus International Collaborating Clinics/American College of Rheumatology
SLICC: Systemic Lupus International Collaborating Clinics
