# Supplementary material for: Performance of the systemic lupus erythematosus risk probability index (SLERPI) in the Egyptian college of rheumatology (ECR) study cohort
Source: Clin Rheumatol. 2024 Nov 4;44(1):207–15. doi: 10.1007/s10067-024-07210-0 (PMC11729068; doi:10.1007/s10067-024-07210-0)

**Supplementary figure 2.** The graph represents the receiver operating curve with a calculated area under the curve indicating an excellent capacity of the SLERPI to discriminate SLE versus miscellaneous rheumatology disease controls after excluding patients with connective tissues diseases.


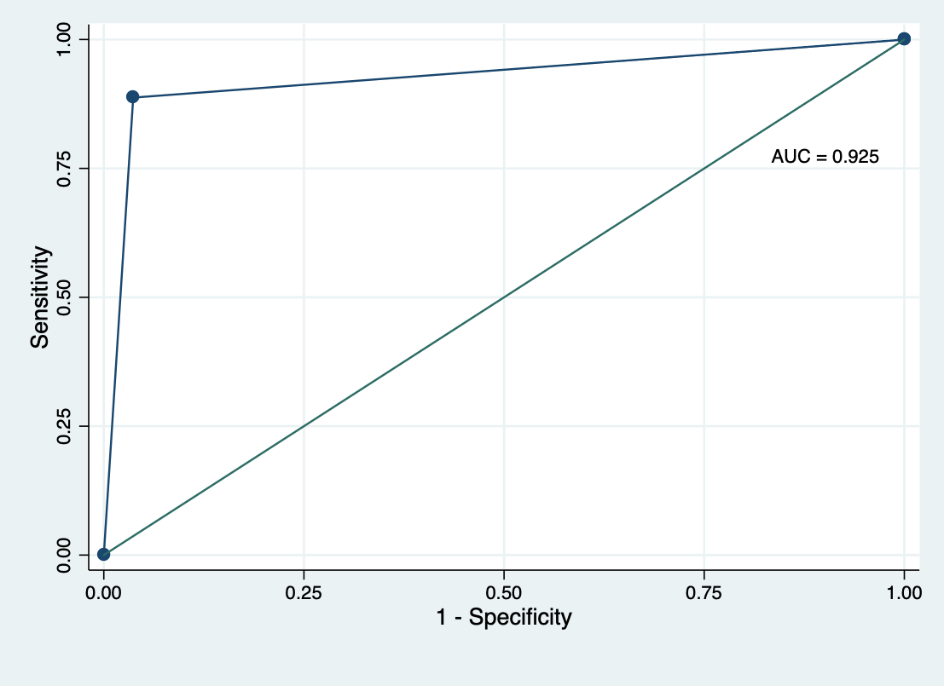

Supplement: Supplementary file 1 — Supplementary file1 (DOCX 82 KB) [file 10067_2024_7210_MOESM1_ESM.docx]
